# Sex-Biased Expression of MicroRNAs in *Schistosoma mansoni*

**DOI:** 10.1371/journal.pntd.0002402

**Published:** 2013-09-12

**Authors:** Antonio Marco, Ana Kozomara, Jerome H. L. Hui, Aidan M. Emery, David Rollinson, Sam Griffiths-Jones, Matthew Ronshaugen

**Affiliations:** 1 Faculty of Life Sciences, Michael Smith Building, Oxford Road, University of Manchester, Manchester, United Kingdom; 2 School of Biological Sciences, University of Essex, Colchester, United Kingdom; 3 Wolfson Wellcome Biomedical Laboratories, Department of Life Sciences, Natural History Museum, London, United Kingdom; University of Queensland, Australia

## Abstract

Schistosomiasis is an important neglected tropical disease caused by digenean helminth parasites of the genus *Schistosoma*. Schistosomes are unusual in that they are dioecious and the adult worms live in the blood system. MicroRNAs play crucial roles during gene regulation and are likely to be important in sex differentiation in dioecious species. Here we characterize 112 microRNAs from adult *Schistosoma mansoni* individuals, including 84 novel microRNA families, and investigate the expression pattern in different sexes. By deep sequencing, we measured the relative expression levels of conserved and newly identified microRNAs between male and female samples. We observed that 13 microRNAs exhibited sex-biased expression, 10 of which are more abundant in females than in males. Sex chromosomes showed a paucity of female-biased genes, as predicted by theoretical evolutionary models. We propose that the recent emergence of separate sexes in *Schistosoma* had an effect on the chromosomal distribution and evolution of microRNAs, and that microRNAs are likely to participate in the sex differentiation/maintenance process.

## Author Summary

Schistosomiasis is the second most common disease caused by a parasite, affecting over 200 million people. The parasites involved are flatworms of the genus *Schistosoma*. Unlike most non-parasitic flatworms, *Schistosoma* species have separate sexes, and the emergence of sex has been associated with the development of a parasitic lifestyle. The identification of gene products that are expressed in a sex-biased fashion permits the study of the origin of sexual dimorphism and, in the case of the schistosomes, the evolution of a human parasite. Here we investigated the differential expression of microRNAs in male and female individuals of the species *Schistosoma mansoni*. MicroRNAs are crucial gene regulators. We observed that many new microRNAs emerged in the evolutionary lineage leading to the schistosomes. However, many sex-biased microRNAs were present in the hermaphrodite ancestor of the flatworms, and therefore acquired sex-biased expression later on. Our results suggest that changes in microRNA expression patterns were associated with the emergence of separate sexes in the schistosomes.

## Introduction

Human schistosomiasis is a neglected tropical disease (NTD) caused by blood flukes of the genus *Schistosoma*. Schistosomiasis is estimated to affect over 200 million people in developing tropical and subtropical countries, with over 90% of cases being confined to Africa [1], [2]. *Schistosoma mansoni* is primarily responsible for intestinal and hepatic schistosomiasis in Africa, the Arabian peninsula, parts of South America and the Caribbean Islands [3]. Unlike most other flatworms (phylum Platyhelminthes), *Schistosoma* species are dioecious; that is, they have two differentiated sexes. The emergence of sexual dimorphism in these species is believed to be associated with adaptation to warm-blooded vertebrates from a hermaphrodite ancestor in cold-blooded vertebrates [4]. *Schistosoma mansoni* has seven pairs of autosomes and one pair of sexual chromosomes with a ZW system, i.e., females are the heterogametic sex [5]. Like other species with a ZW-based system of sex determination, there is no apparent global dosage compensation in females [6].

The origin of *Schistosoma* sexuality has attracted much attention [4], [7]–[9]. Moreover, as the eggs laid by the female worms are primarily responsible for the pathology associated with schistosomiasis, the mechanisms associated with pairing and egg-laying, including expression of sex specific genes are of great interest. In the last years, different groups have characterized, using genomic and proteomic approaches, gene products with differential expression between males and females in *Schistosoma* species [10]–[13]. Since both sexes are necessary for the colonization of the host, sex-biased genes are potential targets for the infection control of schistosomes.

MicroRNAs are short endogenous RNA molecules that regulate gene expression by targeting mature mRNA transcripts [14]. This mechanism of post-transcriptional regulation is conserved in animals and is likely to be involved in all aspects of cellular function [15]. The microRNA content of *Schistosoma japonicum*, which affects large endemic areas around the river Yangtze in China, has been studied in detail by several groups [16]–[21]. In addition, the previous characterization of microRNAs from other non dioecious species of flatworms, such as *Echinococcus granulosus and* the non-parasitic *Schmidtea mediterranea* [22], [23], provides a background against which to identify *Schistosoma*-specific microRNAs with a potential role in sexual development and host-parasite interactions.

Current knowledge of *S. mansoni* microRNAs is limited and mostly based on computational predictions [24], [25]. Moreover, *S. mansoni* provides an excellent model to study the evolution and function of sex-biased microRNAs. Here we use deep sequencing of RNA libraries to explore the microRNA content of *S. mansoni*, identify microRNAs specific to the schistosomes, and study the potential impact of sex-biased microRNAs in sexual differentiation.

## Results

### The microRNAs of *Schistosoma mansoni*

We have used small RNA deep sequencing to identify a total of 112 microRNAs in adult *S. mansoni* (Table 1, Supplementary Files S1 and S2). Valid microRNA candidates were required to have reads mapping to both arms of the precursor sequences (representing mature microRNA and microRNA* sequences) except for those with a previously validated homolog (see Materials and Methods). Our microRNA annotation procedure was intentionally conservative: we may not have detected some *bona fide* microRNAs, but our predictions are of high confidence.

**Table 1:** MicroRNAs in *Schistosoma mansoni*.

	Read count			Read count			Read count	
MicroRNA	SO	MS	MicroRNA	SO	MS	MicroRNA	SO	MS
***Chromosome 1***			***Chromosome 3***			***Chromosome 7***		
sma-mir-219	5541	1608	sma-mir-8430	34	0	sma-mir-8454	137	0
sma-mir-8	119	9298	sma-mir-8431	57	0	sma-mir-8455	23	0
sma-mir-8403	61	1	sma-mir-8432	53	0	sma-let-7	13605	11621
sma-mir-8404	23	0	sma-mir-8433	24	0	sma-mir-8456	38	0
sma-mir-125c	16027	25268	sma-mir-8434	58	0	sma-mir-8457	359	25
sma-mir-125a	96750	159980	sma-mir-8435	379	0	sma-mir-8458	17	32
sma-mir-8405	253	0	sma-mir-8436	33	0			
sma-mir-36a	16334	26988	sma-mir-8437	515	2411	***Chromosome Z/W***		
sma-mir-36b	25591	4404	sma-mir-8438	215	0	sma-mir-1a	6102	1028
sma-mir-8406	668	0	sma-mir-8439	91	0	sma-mir-755	12259	29358
sma-mir-8407	360	0				sma-mir-8459	27	0
sma-mir-8408	1526	0	***Chromosome 4***			sma-mir-96	17249	234
sma-mir-8409	22	0	sma-mir-8440	142	0	sma-mir-8460	578	0
sma-mir-8410	19	0	sma-mir-8441	28	0	sma-mir-8461	38	0
sma-mir-8411	301	0	sma-mir-745	93335	1888	sma-mir-1b	6220	1162
sma-mir-190	3591	4938	sma-mir-8442	136	0	sma-mir-8462	120	0
sma-mir-8412	18	0	sma-mir-10	52115	104344	sma-mir-8463	23	0
sma-mir-8413	36	0	sma-mir-281	2903	1114	sma-mir-8464	17	0
sma-mir-8414	133	0	sma-mir-8443	89	0	sma-mir-8465	564	0
sma-mir-8415	49	0	sma-mir-8444	59	0	sma-mir-8466	497	0
sma-mir-8416	53	0	sma-mir-7	18242	82	sma-mir-71a	24483	37197
sma-mir-8417	272	0	sma-mir-8445	52	0	sma-mir-2a	4176	8707
sma-mir-8418	900	0	sma-mir-8446	21679	0	sma-mir-2b	18940	10956
sma-mir-8419	37	0				sma-mir-2e	7426	3608
sma-mir-8420	26	0	***Chromosome 5***			sma-mir-8467	278	0
			sma-mir-2c	1459	1550	sma-mir-8468	52	0
***Chromosome 2***			sma-mir-2d	644	924	sma-mir-8469	192	0
sma-mir-8421	101	0	sma-mir-2f	582	722	sma-mir-8470	16	0
sma-mir-8422	66	0	sma-mir-71b	2374	3860			
sma-mir-8423	1905	0	sma-mir-8447	102	1484	***Unplaced***		
sma-mir-8424	288	0				sma-mir-8471	248	0
sma-mir-8425	34	0	***Chromosome 6***			sma-mir-8472	74	0
sma-mir-8426	61	0	sma-mir-8448	88	0	sma-mir-2162	6486	16531
sma-mir-8427	39	0	sma-mir-31	117	14	sma-mir-8473	355	0
sma-mir-8428	21	1	sma-mir-8449	25	0	sma-mir-8474	14	0
sma-mir-8429	73	1	sma-mir-8450	297	0	sma-mir-8475	1078	0
sma-mir-61	21357	24648	sma-mir-8451	28	0	sma-mir-8476	183	0
			sma-mir-124	7978	1084	sma-bantam	18135	175657
			sma-mir-8452	293	0	sma-mir-8477	25	0
			sma-mir-8453	68	0	sma-mir-8478	39	0
			sma-mir-124b	61	0	sma-mir-8479	112	0
						sma-mir-8480	74	0
						sma-mir-8481	597	0
						sma-mir-8482	52	0
						sma-mir-8483	21	0
						sma-mir-8484	187	0

SO: AB SOLiD sequencing; MS: Illumina MiSeq sequencing.

De Souza Gomes *et al.* [25] computationally identified 42 microRNA loci in *S. mansoni*, significantly expanding the previous set of 6 microRNAs [16], [24]. We confirmed 20 of these (Table 1), all conserved in other species. We failed to detect the remaining 23. All but two of the unconfirmed microRNAs were not conserved in other flatworms. A second recent work characterized 211 novel microRNAs in *S. mansoni* by cloning of small RNA sequences from adults and schistosomulas [26]. However, the majority of the candidate microRNAs map to many positions in the genome and only a few are reported to be within putative precursor hairpin structures. Indeed, only two of the reported 211 microRNAs were confirmed in our analyses (mir-71a and let-7). We identify 92 microRNAs not previously annotated in *S. mansoni*, eight with obvious homologs in other species (Table 1). Amongst these, we show that the deeply-conserved mir-124 locus produces microRNAs from both genomic strands in *S. mansoni* (Supplementary Files S2). The remaining 84 novel predictions had no detectable similarity with any known microRNA.

**Figure 1:**
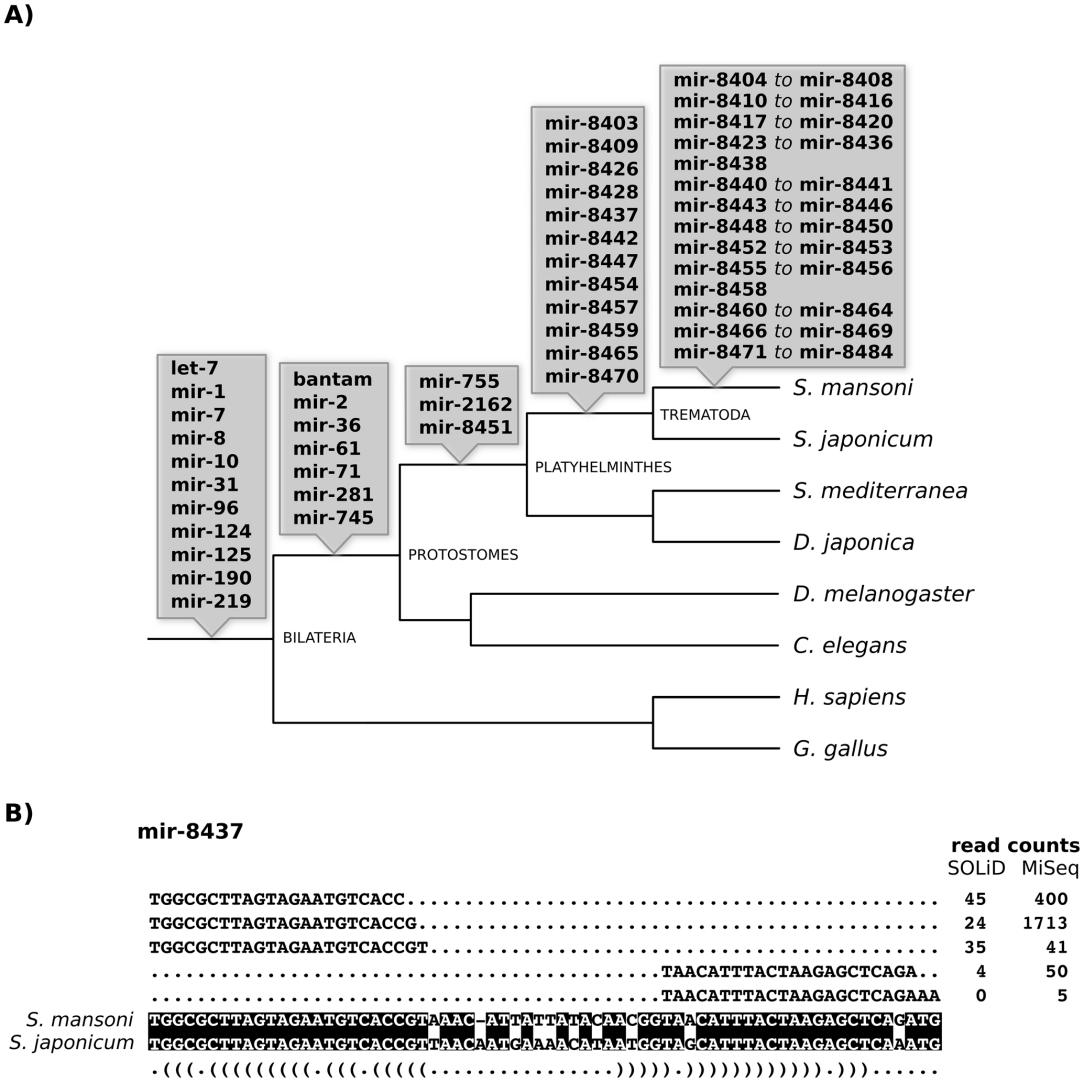
The origin of microRNAs of *Schistosoma mansoni*. A) MicroRNAs characterized from small RNA libraries and their evolutionary origin based on homology searches. B) Example of a newly discovered microRNA in *S. mansoni* that is also conserved in *S. japonicum*. Conserved nucleotides between the two species are shown with a black background. The number of uniquely mapped reads for each sequence in *S. mansoni* is shown in the right, for both SOLiD and MiSeq datasets.

To characterize the microRNAs conserved in the parasitic *Schistosoma* genus, we compared our sequenced microRNAs with those already described for *S. japonicum* [17]–[19], [21]. We found that 26 out of our 112 microRNAs were conserved between these two species (Figure 1). In order to determine how many of those 26 are specific to the *Schistosoma* lineage, we searched the genomes of the flatworms *Schmidtea mediterranea* [22] and *Dugesia japonica* [27] for homologous sequences. Strikingly, all known microRNAs conserved between *Schistosoma* species were also conserved in other flatworms. We also detected a set of *S. japonicum* homologs for 12 of our newly identified *S. mansoni* sequences that were not conserved in other platyhelminthes (Figures 1A and B). Hence, these 12 microRNAs are the first instances of schistosome-specific microRNAs.

Homology searches in other animals showed that three microRNAs are specific to platyhelminthes: mir-755, mir-2162 and mir-8451 (Figure 1A). Thirteen microRNAs are protostome-specific and the remaining 10 are conserved across the animal kingdom (Figure 1A). A total of 71 microRNAs identified in this study are not detected in any other species, and are therefore likely to be *S. mansoni* specific.

### Sex-biased expression of microRNAs in *S.mansoni*

**Figure 2:**
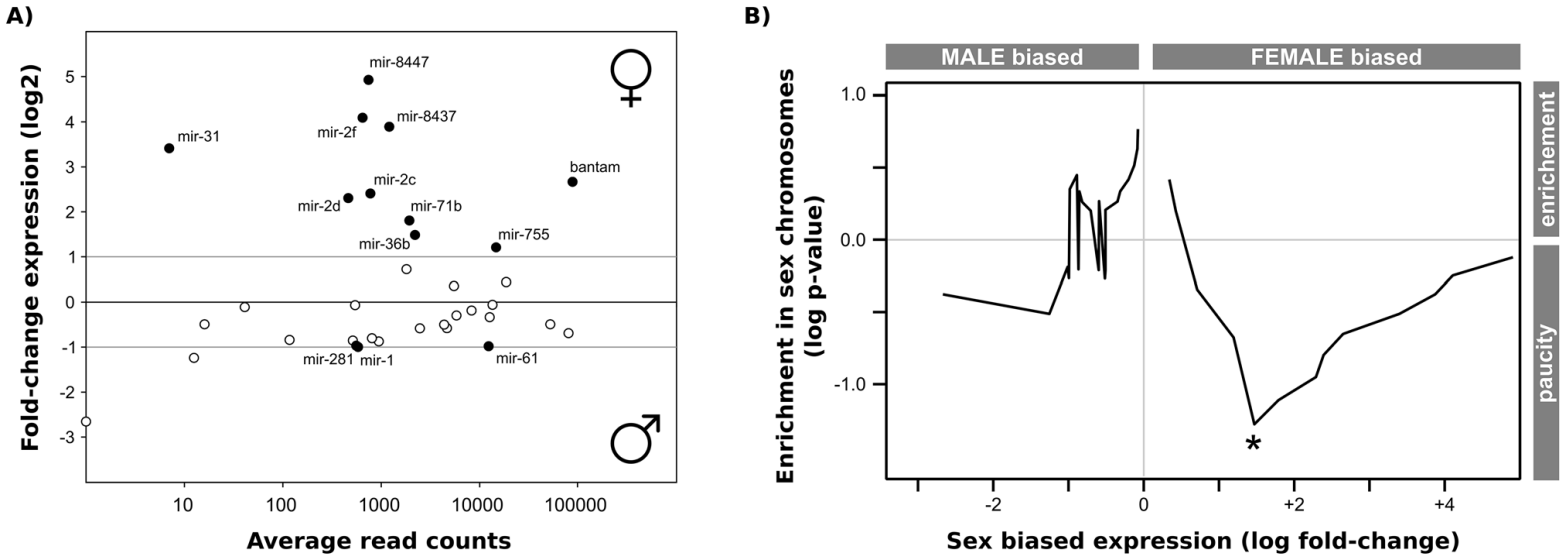
Sex-biased expression of microRNAs in *S.* *mansoni*. A) Smear plot of normalized expression levels (log fold-change) for microRNAs in male and female samples against the total read counts (log average count). Black line indicates equal expression in both sexes. Grey lines represent 2-fold changes between samples. Filled circles are microRNAs with a significant expression fold change between sexes (see Materials and Methods). B) Relative enrichment/paucity of sex-biased microRNAs in the sex chromosome (ZW). Each expression level fold-change threshold (x-axis) is plotted against the logarithm of the p-value (Fisher's exact test) for enrichment or paucity in the sex chromosome.

To explore potential sex-biased expression, we compared the relative expression levels of the 112 microRNAs in males and females (Figure 2A). We found 13 microRNAs that are differentially expressed between males and females. A significant excess [10] show increased expression in females (Figure 2A, Table 2). We further quantified the relative expression level of all 3 male-biased and 4 of the female-biased microRNAs by real-time PCR (see Materials and Methods). The observed fold changes in our qPCR experiments were consistent with those observed in our RNAseq analysis (Table 3), although the two least biased microRNAs by RNAseq show small and non-significant changes by qPCR.

**Table 2:** MicroRNAs with sex-biased expression.

Male-biased expression			Female-biased expression		
microRNA	logFC male/female^1^	q-value^2^	microRNA	LogFC female/male^1^	q-value^2^
mir-1b	1.0	0.064	mir-8447	4.9	<0.001
mir-61	1.0	0.061	mir-2f	4.1	<0.001
mir-281	1.0	0.071	mir-8437	3.9	<0.001
			mir-31	3.4	0.006
			bantam	2.7	<0.001
			mir-2c	2.4	<0.001
			mir-2d	2.3	<0.001
			mir-71b	1.8	<0.001
			mir-36b	1.5	0.002
			mir-755	1.2	0.014

1 Log Fold Change after Upper Quartile normalization of Illumina MiSeq read counts.

2 False discovery rate (q-value) for a dispersion of 0.2 according to edgeR manual (see Methods).

**Table 3:** Real-time PCR validation of sex-biased expressed microRNAs.

	microRNA	qPCR logFC^1^	p-value^2^
**male-biased**	mir-1b	0.7	0.179
	mir-61	1.4	0.008
	mir-281	0.1	0.493
**female-biased**	mir-8437	4.0	<0.001
	bantam	2.5	<0.001
	mir-71b	1.7	0.022
	mir-36b	0.2	0.253

1 Log Fold Change for the differences between Ct values for target and control microRNAs.

2 P-values based on t-test of Ct value differences for three technical replicates.

We next evaluated whether microRNA loci on the sex chromosomes are biased towards differential expression between sexes. The current assembly of the *S. mansoni* genome does not differentiate between Z and W chromosomes. At this stage, we cannot therefore evaluate the two sexual chromosomes separately. In Figure 2B, we plot the relative enrichment of sex-chromosome-linked microRNAs for male and female-biased expression. We observed that the sex chromosome has fewer female-biased microRNAs (∼3-fold change) than expected by chance, although the difference is only marginally significant (p = 0.10). The data therefore indicate that microRNA genes with female sex-biased expression may have a tendency to move out of sex chromosomes (see Discussion).

The mir-71/mir-2 microRNA cluster is highly conserved in invertebrates, and it has been shown that this cluster is duplicated in Platyhelminthes [25], [28]. Interestingly, one of the clusters (mir-71/2a/2b/2e) is on the sex chromosomes while the other (mir-71b/2f/2d/2c) is on chromosome 5 [25], [29]. This has led some authors to postulate that the mir-71/mir-2 clusters may be involved in sexual maturation in *Schistosoma* [25]. Our analysis reveals that all microRNAs in the autosomal cluster have female-biased expression, while the sex chromosome cluster does not show any bias (Figure 2A). Interestingly, the two clusters emerged by a duplication in the ancestral lineage leading to *Schistosoma*, and the multiple copies in other Platyhelminthes [29], [30] came from independent duplication events (Supplementary Files S4). This example may shed some light on how sex chromosomes evolved in dioecious species (see Discussion).

## Discussion

Although the computational prediction of microRNAs has been useful to understand the biology of small RNAs in *S. mansoni* [25], sequencing is required to validate the existence of these microRNAs as well as for detecting new sequences. Our work has confirmed the existence of 20 of the microRNAs predicted by de Souza Gomes et al. [25] and we have expanded the *S. mansoni* microRNA set to 112 loci. We specifically detect microRNAs expressed in sexually mature adults, and microRNAs specifically expressed in other developmental stages (such as schistosomulas) may have escaped our analysis. The use of deep sequencing also permits the characterization of microRNAs produced from both strands of the same locus, and we have identified sense and antisense microRNA production from the mir-124 locus. However, there is no evidence that this microRNA is also bidirectionally transcribed in other species. Indeed, bidirectionally transcribed microRNAs are rare and poorly conserved; only two cases of conserved bidirectional microRNAs are known in protostomes: iab-4 and mir-307 [31].

We observe an excess of microRNAs that exhibit female-biased expression (Figure 2A). This is in agreement with the overall female-bias observed for protein-coding genes in both *S. mansoni* [10] and *S. japonicum* [11], although a recent expression analysis in *S. japonicum* showed no gender bias [13]. Recently, a work in the parasitic nematode *Ascaris suum* showed that microRNAs are differentially expressed between males and females [32]. Although the differences were small and the targeting properties of male and female microRNAs similar, they reported a tendency of male microRNA to target extracellular proteins [32]. Another work in the bird *Taeniopygia guttata* (zebra finch), suggests that the male-biased expressed microRNA mir-2954 specifically target genes in the Z chromosome, and may be involved in sexual dimorphism in song behavior [33]. Together, these papers and our work point to a general mechanism of microRNA-modulation of sex-specific gene expression.

A recent work shows that some microRNAs are specifically expressed in *Schistosoma japonicum* eggs [21]. In that work, the authors also measured the microRNA expression levels in males and females. We reanalyse their data and find that two out of our three male-biased microRNAs (mir-1 and mir-61) have a consistent bias in *Schistosoma japonicum*, while the third is not present in their dataset. Also, five of our female-biased microRNAs also showed a female bias in their work (mir-71b, mir-2c, mir-2d, bantam and mir-31). These findings further validate our results and show that the sex-biased pattern of microRNA expression is evolutionarily conserved between these two species.

Sex-biased gene expression affects the genetic composition of chromosomes, since selection has different effects on sex-biased genes depending on whether they are located on sex chromosomes or on autosomes (reviewed in [34]). Likewise, sex chromosomes have distinctive evolutionary patterns, which affect the genes encoded within [35]. We may therefore expect to see signatures of chromosome evolution in sex-biased microRNAs. Indeed, we observed that microRNAs that are female sex-biased are depleted in the sexual chromosomes (Figure 2B). This may indicate a loss of sex-biased genes at the sex chromosomes. One interesting example is the female-biased mir-71b/2f/2c/2d autosomal microRNA cluster, which has a paralogous copy in the sex chromosome with no biased expression. If a microRNA gene that is selectively advantageous for females becomes part of a sex chromosome (by sexualisation of the chromosome, or otherwise), selection over this gene will be less efficient in the heterogametic sex (females in our case), generating a conflict between expression pattern and chromosomal location. Duplication of a gene into an autosome has been recently proposed as a way to escape such conflict (reviewed in [36]), and the mir-71/mir-2 cluster duplication appears to be an example of this. Although a more comprehensive analysis of duplicated microRNAs with sex-biased expression is required to confirm this, our analysis shows that sex-biased expressed microRNAs have an impact in shaping the genome during evolution.

The study of microRNAs in the schistosomes is of both evolutionary and biomedical interest. First, the recent evolution of a sexual reproductive system from a hermaphrodite ancestor can give clues about how sexuality emerged in other species. Our data are consistent with the acquisition of sex-biased expression of conserved microRNAs soon after the species become dioecious. Second, the characterization of *Schistosoma*-specific microRNAs may provide new targets for infection control. In this work, we characterize for the first time 12 microRNAs conserved between *S. mansoni* and *S. japonicum* but not in other platyhelminthes (nor in other animals). Two of these sequences also showed female-biased expression (mir-8437 and mir-8447). However, some important questions remain to be answered: Are microRNAs conserved in the two studied schistosomes also conserved in other *Schistosoma* species or in other trematodes? Is sex-biased expression of microRNAs associated with sex-biased expression of their targets? Do other dioecious flatworms have sex-biased expression of microRNAs? The genomic sequencing of more platyhelminthes and characterization of their microRNAs will help us to answer these questions.

## Materials and Methods

### Small RNA extraction, library construction and sequencing

For the detection of expressed microRNAs, female mice (BKW strain) were infected with Belo-Horizonte strain *Schistosoma mansoni* parasites by paddling in water containing 200 cercariae. Seven weeks after infection, adult schistosomes were collected from the mice post-mortem by hepatic portal perfusion. RNA was extracted from adult schistosome samples with the miRVana miRNA isolation kit (Ambion). We used two sequencing technologies, AB SOLiD and Illumina MiSeq, to sequence *S. mansoni* small RNA libraries. The extremely deep coverage provided by SOLiD sequencing provides high sensitivity for the discovery of novel microRNAs. We further used Illumina MiSeq sequencing of gender-specific libraries to compare the expression level of microRNAs between males and females. Library construction was performed as previous described [37] using the SOLiD Small RNA Expression Kit (Ambion). SOLiD sequencing was performed at the Center for Genomic Research at the University of Liverpool. We obtained a total of 124,341,126 SOLiD sequence reads from two libraries. For the gender-specific differential expression of microRNAs, we prepared RNA libraries with the miRVana kit from separate male and female samples (provided by Andrew MacDonald and Rinku Rajan at the University of Edinburgh), and prepared libraries for Illumina MiSeq sequencing according to the manufacturer's instructions. MiSeq samples were sequenced in the Genomics Core Facility at the University of Manchester. High-throughput datasets were deposited in Gene Expression Omnibus (GEO) at NCBI (accession number: GSE49359).

### MicroRNA detection and annotation

Sequencing reads from male and female libraries were separately mapped to the *S. mansoni* reference genome (assembly 5.1 available at http://www.genedb.org/Homepage/Smansoni; [38], [39]) with Bowtie 0.12 using the sequential trimming strategy implemented in SeqTrimMap 1.0 [40] allowing 2 mismatches. Sequences mapping to potential rRNAs or tRNAs were first removed. Putative tRNAs were predicted in the genome sequence with tRNAscan-SE using default parameters [41] and ribosomal RNAs (rRNAs) were extracted from the SILVA database (http://www.arb-silva.de/, release 108). Mapped reads with a length of 19–25 nucleotides, matching five or fewer positions in the genome (a total of 63,771,124 sequence reads), were used to detect microRNAs as previously described [37], [40]. We further used BLAST [42] to search microRNA candidates against the *S. mansoni* genome and discarded those with more than 5 hits (E-value <e-10, 80% query coverage) to remove potential repetitive elements: 44 candidate sequences did not pass this filter. MicroRNA candidates were manually inspected. Potential homologs of known microRNAs (detected by BLASTN against all hairpin sequences from miRBase version 17 [43]) with reads mapped from our datasets but which did not pass our criteria were also retained. Homolog of our microRNA candidates were predicted in the genome sequences of *S. japonicum* (version 2), *S. mediterranea* (v. 3.1), *Caenorhabditis elegans* (v. 7.1), *Tribolium castaneum* (v. 3.0), *Drosophila melanogaster* (v. 5.1), *Homo sapiens* (v. 37.1) and *Gallus gallus* (v. 2.1), with parameters: −W 4, −r +2, −q −3. Only sequences with a predicted hairpin structure and conserving at least one mature sequence were considered as putative homologs.

### Differential expression analyses

We mapped reads produced from MiSeq sequencing reactions to our annotated *S. mansoni* microRNAs and discarded all reads that map to more than one microRNA locus. Read counts were transformed with the ‘upper quartile’ normalization using the edgeR package [44], following the suggestions in [45]. Other normalization procedures (‘TMM’, ‘LOWESS’ and ‘no normalization’) did not change the results (Supplementary Files S3). Fold changes in expression levels are given in logarithms in base 2. We consider a dispersion of expression between experiments of 0.2 and a false discovery rate of 10%. With these parameters, microRNAs showing a two-fold difference in their expression levels are considered to be sex-biased, as routinely suggested [46], [47]. The expression level in males and females of seven microRNAs (mir-1b, mir-61, mir-281, mir-36b, mir-71b, bantam and mir-8437) were further validated by quantitative PCR. We used custom made TaqMan assays manufactured by Life Technologies. Fluorescent quantification was done in a Chromo 4 qPCR system (BioRad) for a log fluorescent threshold of 0.05, using mir-36a (the microRNA showing the least bias in the MiSeq experiments) as the non-sex-biased control microRNA. For each amplification, we performed three technical replicates to estimate the significance of the observed differences.

### Ethical statement

Laboratory animal use was within a designated facility regulated under the terms of the UK Animals (Scientific Procedures) Act, 1986, complying with all requirements therein. The experiments involving mice in this study were approved by the Natural History Museum Ethical Review Process and work was carried out under Home Office project licence 70/6834.

## Supporting Information

File S1 Sequences, chromosomal location and number of reads for all *Schistosoma mansoni* microRNAs characterized in this study.
(XLS)

File S2 Structural features of *Schistosoma mansoni* microRNA precursors.
(TXT)

File S3 Differential expression analysis between male and female *Schistosoma mansoni* specimens for different normalization methods.
(XLS)

File S4 Phylogenetic relationships between mir-71 sequences in *Schistosoma* mansoni, *S. japonicum* and *Schmidtea mediterranea*.
(PDF)
