# Supplementary material for: Sex-Biased Expression of MicroRNAs in Schistosoma mansoni
Source: PLoS Negl Trop Dis. 2013 Sep 12;7(9):e2402. doi: 10.1371/journal.pntd.0002402 (PMC3772069; doi:10.1371/journal.pntd.0002402)
Supplement: File S4 — Phylogenetic relationships between mir-71 sequences in Schistosoma mansoni, S. japonicum and Schmidtea mediterranea. (PDF) [file pntd.0002402.s004.pdf]

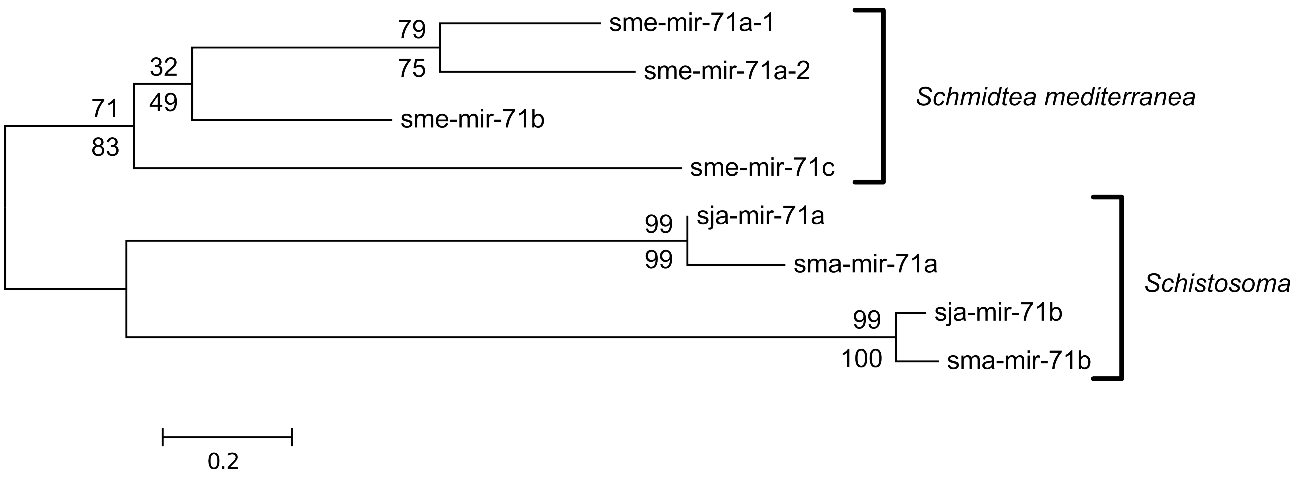

Supplementary file 4. Phylogenetic relationships between mir-71 microRNAs in *Schistosoma* and *Schmidtea*. MicroRNA precursor sequences were aligned with ClustalX 2 [1] and phylogentic trees built with MEGA 5 [2] using the Maximum-Likelihood (ML) and Neighbor-Joining (NJ) routines are recommended. The tree in the figure shows the topology of the NJ tree and the bootstrap percentages for NJ (above each branch) and ML (below each branch).

References

1. Larkin MA, Blackshields G, Brown NP, Chenna R, McGettigan PA, et al. (2007) Clustal W and Clustal X version 2.0. Bioinforma Oxf Engl 23: 2947–2948.

2. Tamura K, Peterson D, Peterson N, Stecher G, Nei M, et al. (2011) MEGA5: Molecular Evolutionary Genetics Analysis Using Maximum Likelihood, Evolutionary Distance, and Maximum Parsimony Methods. Mol Biol Evol 28: 2731–2739.
